# Use of the Walking Impairment Questionnaire and Walking Estimated-Limitation Calculated by History questionnaire to detect maximal walking distance equal to or lower than 250 m in patients with lower extremity arterial disease

**DOI:** 10.3389/fcvm.2023.968213

**Published:** 2023-03-21

**Authors:** Quentin Tollenaere, Antoine Métairie, Estelle Le Pabic, Alexis Le Faucheur, Guillaume Mahé

**Affiliations:** ^1^Vascular Medicine Unit, Centre Hospitalier Universitaire de Rennes, Rennes, France; ^2^CHU Rennes, Inserm, CIC 1414 (Clinical Investigation Center), Rennes, France; ^3^Univ Rennes, M2S – EA 7470, Rennes, France

**Keywords:** lower extremity artery disease, peripheral artery disease, intermittent claudication, walking distance assessment, questionnaire, treadmill test

## Abstract

**Objective:**

The objective was to assess the accuracy and optimal threshold of the Walking Impairment Questionnaire (WIQ) and the Walking Estimated-Limitation Calculated by History (WELCH) questionnaire in identifying patients with a maximal walking distance (MWD) below or equal to 250 m.

**Methods:**

This retrospective study screened 388 consecutive patients with suspected symptomatic lower extremity arterial disease (LEAD). Collected data included the patient's history, resting ankle-brachial index, WIQ, and WELCH. MWD was assessed with a treadmill test at 2 mph (3.2 km/h) with a 10% grade. An optimized threshold for detection of MWD ≤ 250 m was determined for each questionnaire *via* receiver operating characteristic (ROC) curves. Subsequently, multivariate analysis was performed to build a new simple score to detect MWD ≤ 250 m.

**Results:**

The study included 297 patients (63 ± 10 years old). With a threshold of ≤ 64%, the WIQ predicted MWD ≤ 250 m with an accuracy of 71.4% (66.2, 76.5%). With a threshold of ≤ 22, the WELCH predicted a treadmill walking distance of ≤ 250 m with an accuracy of 68.7% (63.4, 74.0%). A new score with only four “yes or no” questions had an accuracy of 71.4% (66.3, 76.6%). Items on this new score consisted of the level of difficulty of walking 1 block, declared maximum walking distance, usual walking speed, and maximum duration of slow walking.

**Conclusion:**

A WIQ score ≤ 64% and a WELCH score ≤ 22 help to predict a walking distance of ≤ 250 m in a treadmill test at 2 mph (3.2 km/h) with a 10% grade. A 4-item score could be used for rapid evaluation of walking distance among patients with LEAD, but the validity of this 4-item score requires further confirmation studies.

## 1. Introduction

Lower extremity arterial disease (LEAD) affects more than 230 million people worldwide (1). Walking distance is closely related to quality of life (QOL), as measured by QOL questionnaires in patients with LEAD. Maximal walking distance (or time) as established during treadmill walking is widely used as an index of walking impairment. The assessment of maximal walking distance (MWD) is also an issue in the Rutherford LEAD classification, where identification of the stage depends on the patient's ability to walk for 5 min at 2 mph at a 10% slope that corresponds to 267 m, rounded to 250 m (2). Vascular surgery societies recommend the Rutherford classification for clinical trials as well as in clinical practice (3). Furthermore, the American Medical Association, health insurance companies and public health regulation agencies, use walking distance to evaluate LEAD impairment for compensation schemes and from the perspective of health economics (4). Therefore, clinical measurements of MWD are useful in the prediction of patients' functional limitations, in the assessment of patients for surgery and endovascular procedures, and for the inclusion of patients in clinical trials.

Clinicians can assess the functional limitations of patients with LEAD by several means: standardized treadmill tests, a 6-min walking test, and more recently, global positioning system (GPS) recordings (5). Standardized treadmill tests are the only validated method for LEAD diagnosis and remain the gold standard. However, standardized treadmill testing is costly. Treadmill testing for angina costs around USD 500 per patient when considering staffing requirements (medical and clerical), equipment (charge per person), and overhead charges (room rental and utility costs), and it requires medical attention for around 20 min. Treadmill testing for LEAD suffers from the same limitations and is less commonly available (6).

A number of questionnaires, such as the Walking Impairment Questionnaire (WIQ) and the Walking Estimated-Limitation Calculated by History (WELCH) questionnaire, are readily accessible and represent tools of interest for estimating patients' impairment (7, 8). However, these questionnaires do not measure MWD and there is no simple known rule to determine a patient's walking distance from the results of such questionnaires. We hypothesize that from among the questions of the WIQ and the WELCH and measures of patient clinical characteristics, a subset of items could be selected that would accurately reflect MWD, with a focus on identifying patients with MWD lower than 250 m. This could be of interest to select patients for clinical trials and to assess the severity of claudication.

Therefore, the primary objective of this study was to determine which cut-off values of WIQ and WELCH scores predict a maximal walking distance equal to or lower than 250 m on the treadmill. The secondary objective was to determine which clinical variables or questions from the WIQ and the WELCH questionnaire best predict a MWD below or equal to 250 m.

## 2. Methods

### 2.1. Type of study

This was a cross-sectional, non-interventional, monocentric study based on a retrospective analysis of consecutive patients referred to the vascular unit of University Hospital, Rennes, France for exertional limb symptoms and suspected of having LEAD.

We recruited all 388 patients between 1 January 2017 and 1 September 2020. All patients consulted for treadmill MWD evaluation with an established or suspected diagnosis of LEAD.

The Exercise PAD cohort study is registered with the American National Institutes of Health database under reference n° NCT03186391. All patients signed an agreement explaining the research protocol and were treated in accordance with the Helsinki convention (9). The protocol was submitted to the local ethics committee of University Hospital of Rennes.

### 2.2. Inclusion and exclusion criteria

Patients were included in the data if they met the hemodynamic criterion for LEAD, i.e., a resting ankle-brachial index (ABI) below or equal to 0.90 or a difference between post-exercise ABI and resting ABI above 18.5% of resting ABI (10). Exclusion criteria were (i) inability to answer the WIQ or the WELCH questionnaire, (ii) interruption of the Strandness Test due to dyspnea or thoracic pain, (iii) interruption of the Strandness Test because the patient could not achieve a speed of 3.2 km/h (roughly 2 mph), and (iv) interruption of the Strandness Test because of purely rheumatologic pain (knee pain, for instance).

### 2.3. Study protocol

Patients underwent a physical examination, medical history (anamnesis), resting ABI measurement, a standard treadmill test, and post-exercise pressure measurements at a single appointment. We collected the following examination data: age; declared walking distance of pain onset; declared maximal walking distance before stopping; and tobacco consumption, graded as past if cessation occurred at least 6 months before examination or as ongoing if cessation occurred < 6 months before or the patient was currently smoking. We also measured weight and height. We completed the patient history on the basis of the patient's current drug prescriptions, the patient's recollection, and previous hospitalization reports or any other data available.

History data included diabetes status, defined by ongoing sugar-lowering treatment or HbA1c > 6.5%; dyslipidemia status, defined by ongoing statin treatment or declared dyslipidemia; hypertension, defined by current use of antihypertensive drugs; presence of vascular graft; presence of vascular stent; history of coronary heart disease or heart stenting or coronary by-pass; history of carotid artery disease, graded as ischemic stroke, transient ischemic stroke, or asymptomatic carotid endarterectomy; and obstructive sleep apnea syndrome.

We asked the patient to spontaneously estimate their own walking distance at pain onset (“declared pain-onset walking distance”, DPWD) and maximal walking distance (“declared maximal walking distance”, DMWD).

We also administered a French version of the WIQ and the WELCH questionnaire to each patient before the exercise.

The Walking Impairment Questionnaire (WIQ) (7) is frequently used to evaluate the impairment of patients with LEAD. A trained physician administered the WIQ to the patients. The WIQ consists of three sets of questions: one regarding the level of difficulty of walking at an average speed, another regarding the level of difficulty of walking 100 m at increasing speeds, and the last regarding the level of difficulty of climbing increasing numbers of flights of stairs. Then, after a specific calculation, all these scores are rounded in the form of percentages and the WIQ score is the average of these three scores. The higher the score, the better the walking capacity of the patient.

The Walking Estimated-Limitation Calculated by History (WELCH) questionnaire (8) is also frequently used to assess the limitations of patients with LEAD. It consists of four questions; the first three relate to the amount of time before stopping at increasing speeds, and the last is a multiplier and compares the patient's speed with that of their relatives. WELCH score varies between 0 and 100. The higher the score, the better the walking capacity of the patient.

Measurement of resting ABI was performed according to American Heart Association recommendations using a hand-held Doppler probe (8 MHz; Basic Atys Medical, Soucieu en Jarrest, France) by a trained vascular medicine physician, with the exception of brachial blood pressure measurements, which were taken using an automated oscillometric blood pressure monitor (Carescape Dinamap V100; GE Healthcare) (4, 11).

The patient was at rest for 10 min in a supine position, relaxed, head and heels supported by an examination desk. We controlled the room temperature at 21 ± 1°C. We used the counterclockwise sequence for pressure measurement: right brachial artery, right posterior tibial artery, right dorsalis pedis artery, left posterior tibial artery, left dorsalis pedis artery, left brachial artery, and right brachial artery. We calculated the ABI by dividing the highest pressure at the lower limb (dorsalis pedis or posterior tibial pressure) by the highest pressure at the arm, as recommended.

We used a treadmill test (3.2 km/h, 10% slope) to determine MWD. Patients were asked to rate their pain on a 0–4 scale, and we stopped the treadmill test if the patient reached 4 on the pain scale or was unable to reach a speed of 3.2 km/h (around 2 mph), in accordance with recommendations (12). We also stopped the treadmill test if the patient experienced acute chest pain or major dyspnea, or when they had completed the 10-min test for a total distance of 525 m. Immediately after the treadmill test, within 1 min, we measured the post-exercise ankle-brachial index, as previously described.

### 2.4. Statistical analysis

Continuous variables are reported in the form mean ± standard deviation (SD) or median, and categorical variables are reported as numbers (percentages).

Receiver Operating Characteristic (ROC) curves were drawn by plotting sensitivity against 1 minus specificity. The optimal threshold was defined as the threshold that maximized sensitivity + specificity, known as the Youden index. For declared walking distances, 250 m was used as a threshold.

Subsequently, we determined the area under the curve (AUC) of the WIQ and WELCH scores, maximal declared walking distance, and declared pain onset walking distance.

We used McNemar tests for 2 by 2 comparisons of the accuracy of the WELCH, the WIQ, and declared walking distance with their respective optimized thresholds.

We calculated the Pearson correlation between WIQ total score and MWD, and between WELCH score and MWD.

Logistic univariate regressions were used to identify the variables associated with MWD. All variables, such as age and sex, were converted to nominal binary variables, using the median value for continuous variables, with the exceptions of body mass index and tobacco consumption. The median value was rounded to the nearest multiple of 50 m for the declared walking distance variable.

For answers to the questionnaire, a step-by-step analysis was used to regroup the answers into groups that maximized AUC. Response options to the WELCH questionnaire were regrouped to create equal-size groups. For the WIQ, a sensitivity analysis was conducted to determine whether the regrouping of the response options into three groups (“no difficulty” in one group, “some difficulty” and “slight difficulty” in a second group, and “much difficulty” and “unable to do” in a third group) had an influence on the outcomes of the statistical analysis.

We did not use composite variables, such as the partial or complete results of the WIQ and combined questions of the WELCH questionnaire, as the aim of the research was not to overcomplicate pre-existing questionnaires. Post-exercise data, such as post-exercise ABI, were not plotted, because the aim of the research was to determine walking distance *ex ante*.

For the ABI, we compared the use of both the median value and a value of 0.90 as thresholds, as the latter is a criterion for LEAD. Both the best ABI and the worst ABI were plotted.

We used logistic univariate regressions over all collected data to identify the variables associated with MWD. We selected a lax *p*-value threshold of < 0.20 to identify variables of interest for further study in a multivariate model. The choice of a loose value of *p* threshold to identify variables associated with MWD allowed more variables to be tested in the multivariate model. Subsequently, a backward stepwise procedure was used to identify explanatory variables. We used a statistical threshold of 0.05 to eliminate variables one by one. We created a score using the multivariate coefficients of the statistically significant variables.

We simplified the score to a 1-digit scoring model by simply rounding the multivariate coefficients to the nearest natural number. Finally, we measured the performance of this model in terms of sensitivity, specificity, and area under the ROC curve.

We adopted a statistical significance threshold of 0.05 for all tests except the univariate model. A 95% confidence interval is reported for all estimates. We used the SAS^®^ 9.4 software package (SAS Institute, Cary, NC, USA) for statistical analysis.

## 3. Results

### 3.1. Patients' demographic characteristics

**Figure 1:**
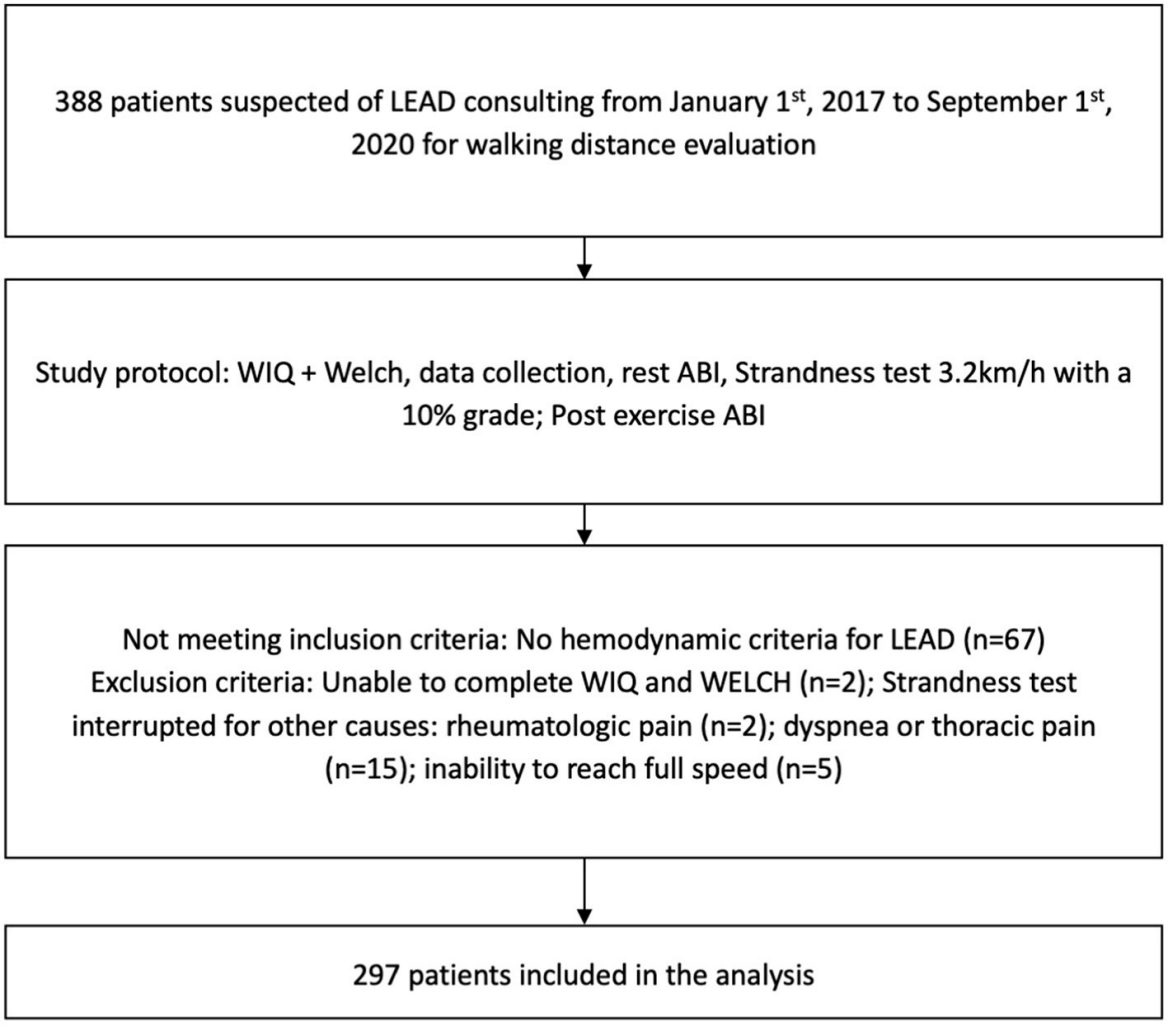
Flow chart with inclusion and exclusion criteria. LEAD, lower extremity arterial disease; WIQ, Walking Impairment Questionnaire; WELCH, Walking Estimated-Limitation Calculated by History; ABI, ankle-brachial index.

We recruited 388 adult patients with suspected LEAD (Figure 1). Sixty-seven patients did not meet the hemodynamic criteria for LEAD and were not included. Twenty-four additional patients were excluded from the analysis because the treadmill test was interrupted for other causes than LEAD, e.g., dyspnea or thoracic pain (*n* = 15), inability to reach full speed (*n* = 5), or purely rheumatologic pain (*n* = 2). These last two patients both experienced knee pain that prevented them from walking and had a history of knee arthrosis. We excluded two additional patients due to missing data, as both were unable to answer the questions of the WIQ and the WELCH questionnaire. In total, we analyzed data from 297 patients.

Among the 297 patients included in the analysis, the mean age was 63 ± 10 years. A total of 83% were men, and the mean body mass index was 27.0 ± 4.4 kg/m^2^. Twenty-six percent of patients had diabetes. Sample characteristics and data collected on a range of variables are detailed in Table 1. The mean WIQ score was 46 ± 25%, and the mean WELCH score was 26 ± 19.

**Table 1:** Population characteristics.

**Variables**	**Population (n=297)**
Age (years)	63.0 ± 10.0
Male sex	247 (83%)
Body mass index (kg/m^2^)	27.0 ± 4.4
DPWD (m)	332 ± 884
DMWD (m)	1,086 ± 2,048
Diabetes	76 (26%)
Dyslipidemia	239 (81%)
Hypertension	233 (79%)
Current smoker or smoking cessation < 6 months	120 (41%)
Smoking cessation > 6 months	141 (48%)
Never smoked	34 (12%)
Lower limb graft or stent	109 (37%)
ACS or coronary stent or bypass	96 (32%)
History of ischemic stroke	29 (10%)
Sleep apnea syndrome	30 (10%)
Worst limb ABI	0.81 ± 0.26
Resting ABI ≤ 0.90	195 (66%)
Best limb ABI	0.94 ± 0.28
WIQ (%)	46 ± 25
WELCH (points)	26 ± 19

Results are presented in the form mean ± standard deviation or number (proportion). ABI, ankle-brachial index; WIQ, Walking Impairment Questionnaire; WELCH, Walking Estimated-Limitation Calculated by History; ACS, acute coronary syndrome; DMWD, declared maximal walking distance; DPWD, declared pain-onset walking distance.

A total of 185 patients did not complete the 250 m walking test; the remaining 112 patients completed it.

### 3.2. Correlations

The correlation coefficient representing correlation with MWD as measured on the treadmill was higher for the WELCH [with a correlation of 0.55 (0.46, 0.62)] and the WIQ [0.51 (0.42, 0.59)] than for the DMWD [with a correlation of 0.41 (0.31, 0.50)] and the DPWD [0.21 (0.10, 0.32)].

### 3.3. Optimized thresholds for detection of 250 m MWD for the WELCH and the WIQ

The AUC of the ROC curves was 0.78 (0.73, 0.83) for the WELCH and 0.74 (0.68, 0.80) for the WIQ. The optimal threshold for detection of a treadmill MWD ≤ 250 m was ≤ 22 for the WELCH and ≤ 64% for the WIQ (Table 2).

**Table 2:** Sensitivity and specificity, area under the receiver operating characteristic curve, AUC, and correlation with measured walking distance for WIQ score, WELCH score, and patients' estimates.

**Score**	**Threshold**	**AUC**	**Sensitivity**	**Specificity**	**Accuracy**	**Correlation**
WELCH	≤ 22	0.78 (0.73, 0.83)	69.7% (63.1, 76.4%)	67.0% (59.8, 74.1%)	68.7% (63.4, 74.0%)	0.55 (0.46, 0.62)
WIQ	≤ 64%	0.74 (0.68, 0.80)	88.1% (83.4, 92.8%)	43.8% (37.3, 50.2%)	71.4% (66.2, 76.5%)	0.51 (0.42, 0.59)
DMWD	≤ 250 m	0.72 (0.66, 0.78)	46.5% (39.3, 53.7%)	76.8% (69.0, 84.6%)	57.9% (52.3, 63.5%)	0.41 (0.31, 0.50)
DPWD	≤ 250 m	0.56 (0.49, 0.63)	73.5% (67.2, 79.9%)	38.4% (31.7, 45.1%)	60.3% (54.7, 65.8%)	0.21 (0.10, 0.32)

AUC, area under the curve; DMWD, declared maximal walking distance; DPWD, declared pain-onset walking distance; WIQ, Walking Impairment Questionnaire; WELCH, Walking Estimated-Limitation Calculated by History. All variables are presented with a 95% confidence interval.

With accuracies of 68.7% (63.4, 74.0%) and 71.4% (66.2, 76.5%) for the WELCH and the WIQ, respectively, using the previously determined thresholds, WIQ and WELCH scores were more accurate for prediction of MWD ≤ 250 m than the patients' declared maximal walking distance (*p* = 0.02) and their declared pain-onset walking distance (*p* < 0.01). There was no statistical difference in terms of accuracy between the WIQ and the WELCH.

### 3.4. Variables associated with walking ≤ 250 m

Among all variables, all questions of the WIQ and the WELCH questionnaire were individually associated with walking ≤ 250 m with *p* < 0.20 and were therefore included for further analysis (Tables 3, 4). As described previously, the response options for each WIQ item were regrouped to form three groups, and the response options for each WELCH item were regrouped to produce similarly sized groups.

**Table 3:** Univariate analysis of questionnaire variables associated with walking ≤ 250 m.

**Variable**	**OR (95% CI)**	***P***
Level of difficulty of walking indoors (WIQ)		*p* = 0.0005
No difficulty	1	
Some difficulty/slight difficulty	3.21 (1.75, 5.89)	
Much difficulty/unable to do	7.39 (0.28, 195.75)	
Level of difficulty of walking 20 m (WIQ)		*p* < 0.0001
No difficulty	1	
Some difficulty/slight difficulty	3.62 (2.02, 6.48)	
Much difficulty/unable to do	7.98 (0.30, 211.63)	
Level of difficulty of walking 50 m (WIQ)		*p* < 0.0001
No difficulty	1	
Some difficulty/slight difficulty	3.32 (2.00, 5.51)	
Much difficulty/unable to do	19.36 (3.47, 108.05)	
Level of difficulty of walking 100 m (WIQ)		*p* < 0.0001
No difficulty	1	
Some difficulty/slight difficulty	2.61 (1.51, 4.50)	
Much difficulty/unable to do	18.96 (6.55, 54.90)	
Level of difficulty of walking 200 m (WIQ)		*p* < 0.0001
No difficulty	1	
Some difficulty/slight difficulty	2.80 (1.47, 5.36)	
Much difficulty/unable to do	9.09 (4.29, 19.27)	
Level of difficulty of walking 300 m (WIQ)		*p* < 0.0001
No difficulty	1	
Some difficulty/slight difficulty	3.38 (1.59, 7.19)	
Much difficulty/unable to do	7.52 (3.57, 15.82)	
Level of difficulty of walking 500 m (WIQ)		*p* < 0.0001
No difficulty	1	
Some difficulty/slight difficulty	4.21 (1.55, 11.39)	
Much difficulty/unable to do	11.27 (4.35, 29.19)	
Level of difficulty of walking one block slowly (WIQ)		*p* = 0.0006
No difficulty	1	
Some difficulty/slight difficulty	2.28 (1.39, 3.76)	
Much difficulty/unable to do	8.85 (1.52, 51.66)	
Level of difficulty of walking one block at average speed (WIQ)		*p* < 0.0001
No difficulty	1	
Some difficulty/slight difficulty	2.09 (1.25, 3.50)	
Much difficulty/unable to do	8.50 (3.17, 22.76)	
Level of difficulty of walking one block quickly (WIQ)		*p* < 0.0001
No difficulty	1	
Some difficulty/slight difficulty	1.85 (0.85, 4.04)	
Much difficulty/unable to do	4.89 (2.23, 10.75)	
Level of difficulty of jogging one block (WIQ)		*p* = 0.0004
No difficulty	1	
Some difficulty/slight difficulty	1.85 (0.43, 7.92)	
Much difficulty/unable to do	5.57 (1.44, 21.58)	
Level of difficulty of climbing one flight of stairs (WIQ)		*p* = 0.0005
No difficulty	1	
Some difficulty/slight difficulty	2.33 (1.41, 3.86)	
Much difficulty/unable to do	4.22 (1.51, 11.76)	
Level of difficulty of climbing two flights of stairs (WIQ)		*p* < 0.0001
No difficulty	1	
Some difficulty/slight difficulty	2.87 (1.63, 5.06)	
Much difficulty/unable to do	5.50 (2.75, 11.02)	
Level of difficulty of climbing three flights of stairs (WIQ)		*p* < 0.0001
No difficulty	1	
Some difficulty/slight difficulty	2.30 (1.10, 4.79)	
Much difficulty/unable to do	6.15 (3.06, 12.36)	
Maximal duration of a slow walk (WELCH)		*p* < 0.0001
“1 hour” or “3 hours or more”	1	
“Not possible,” “30 seconds,” “1 minute,” “3 minutes,” “10 minutes,” or “30 minutes”	5.35 (3.05, 9.39)	
Maximal duration of a walk at a normal pace (WELCH)		*p* < 0.0001
“30 minutes,” “1 hour,” or “3 hours or more”	1	
“Not possible,” “30 seconds,” “1 minute,” “3 minutes,” or “10 minutes”	6.34 (3.63, 11.05)	
Maximal duration of a walk at a rapid pace (WELCH)		*p* < 0.0001
“10 minutes,” “30 minutes,” “1 hour,” or “3 hours or more”	1	
“Not possible,” “30 seconds,” “1 minute,” or “3 minutes”	4.99 (2.91, 8.54)	
Walking speed compared to relatives of same age (WELCH)		*p* < 0.0001
“A bit slower,” “same speed,” or “faster”	1	
“Much slower” or “moderately slower”	3.97 (2.40, 6.57)	

OR, odds ratio; CI, confidence interval; WIQ, Walking Impairment Questionnaire; WELCH, Walking Estimated-Limitation Calculated by History.

**Table 4:** Univariate analysis of variables other than items of the WIQ and WELCH.

**Variable**	**OR (95% CI)**	***P***
Age		*p* = 0.2959
≤ 66 years old	1	
>66 years old	1.30 (0.80, 2.12)	
Sex		*p* = 0.7844
Male	1	
Female	1.09 (0.58, 2.05)	
Body mass index (BMI)		*p* = 0.9785
< 25 kg/m^2^	1	
25–30 kg/m^2^	1.06 (0.61, 1.82)	
≥30 kg/m^2^	1.05 (0.56, 1.96)	
DPWD (declared pain-onset walking distance)		*p* = 0.0021
≥400 m	1	
< 400 m	2.40 (1.37, 4.19)	
DMWD (declared maximal walking distance)		*p* < 0.0001
≥750 m	1	
< 750 m	5.05 (2.98, 8.56)	
Diabetes		*p* = 0.0371
No	1	
Yes	1.83 (1.04, 3.24)	
Treated dyslipidaemia		*p* = 0.3866
Yes	1	
No	1.31 (0.71, 2.40)	
Treated hypertension		*p* = 0.2614
Yes	1	
No	0.72 (0.41, 1.27)	
Tobacco consumption		*p* = 0.6373
Never	1	
Yes, active	1.01 (0.46, 2.25)	
Previous (>6 months)	0.80 (0.37, 1.75)	
Vascular graft or stenting or other vascular surgery of lower limbs		*p* = 0.0062
No	1	
Yes	2.04 (1.22, 3.39)	
NSTEMI, STEMI, or coronary heart disease or ischemic or transient stroke or carotid endarterectomy		*p* = 0.8078
No	1	
Yes	1.06 (0.65, 1.72)	
Resting ankle-brachial index		*p* = 0.0168
One side or none ≤ 0.90	1	
Both sides ≤ 0.90	1.82 (1.11, 2.97)	
Best ankle-brachial index		*p* = 0.0006
≥0.98	1	
< 0.98	2.32 (1.43, 3.76)	
Worst ankle-brachial index		*p* = 0.0033
≥0.90	1	
< 0.90	2.07 (1.27, 3.36)	

NSTEMI, non-ST elevation myocardial infarction; STEMI, ST elevation myocardial infarction; OR, odds ratio; CI, confidence interval; WIQ, Walking Impairment Questionnaire; WELCH, Walking Estimated-Limitation Calculated by History.

In addition to these questionnaire items, the variables declared maximal walking distance, declared pain-onset walking distance, history of vascular graft or stenting or other vascular surgery of lower limbs, diabetes, resting ABI bilaterally ≤ 0.90, worst ABI, and best ABI were associated with walking ≤ 250 m with a *p*-value below 0.20; all these variables were therefore included in the multivariate analysis.

In contrast, age above 66 years, female sex, obesity or overweight, dyslipidemia, hypertension, tobacco consumption, and coronary heart disease were not found to be significantly associated with walking ≤ 250 m.

### 3.5. Explanatory variables

All variables significantly associated with walking ≤ 250 m with *p* ≤ 0.20 were included in a multivariate model with step-by-step analysis. This multivariate analysis showed that a subset of four items could be selected to predict MWD ≤ 250 m accurately, with a high significance value (*p* ≤ 0.01). Results and odds ratios are shown in Table 5.

**Table 5:** Explanatory variables for 250 m treadmill test [odds ratios are presented in the form: odds ratio (95% confidence interval)].

**Explanatory variable**	**Odds ratio (95% CI)**	**Score**	***P*-value**
During the last week, how difficult was it for you to walk 1 block, on level ground, at average speed without stopping to rest?^a^			0.006
No difficulty	1	0	
Some difficulty/slight difficulty	1.14 (0.58, 2.26)	0.13	
Much difficulty/unable to do	6.39 (1.90, 21.50)	1.85	
What maximum distance can you walk before stopping because the pain becomes unbearable?			0.007
Answer strictly above 750 m	1	0	
Answer below or equal to 750 m	2.39 (1.27, 4.49)	0.87	
Compared to the usual walking speed of your relatives, friends, or people of your own age, do you think that you personally usually walk…^b^			0.010
“A bit slower,” “same speed,” or “faster”	1	0	
“Much slower” or “moderately slower”	2.11 (1.20, 3.73)	0.75	
How long can you walk slowly (slower than the usual speed of relatives, friends, or other people of your own age) on level ground without stopping?^b^			0.006
“1 hour” or “3 hours or more”	1	0	
“Not possible,” “30 seconds,” “1 minute,” “3 minutes,” “10 minutes,” or “30 minutes”	2.59 (1.32, 5.09)	0.95	
Threshold when adding variables		>1.95	

a From the WIQ (Walking Impairment Questionnaire).

b From the WELCH (Walking Estimated-Limitation Calculated by History).

CI, confidence interval.

Each explanatory variable was a WIQ or WELCH item with some answers regrouped. Neither pre-existing conditions nor risk factors such as age, diabetes, or smoking were identified as explanatory variables in our analysis.

The maximizing threshold for the calculated values was 1.95. We detail each variable of this intermediate score in Table 5. The higher the score, the greater the risk of walking ≤ 250 m. The distribution of the score according to the treadmill walking distance is presented in the Appendix.

### 3.6. Simplified scoring model

We simplified the score by setting a 1-digit scoring model, as shown in Table 6, with the values rounded to the nearest natural number. The optimal threshold for the 1-digit score was 2. Therefore, we declared patients with a simplified score of 3, 4, or 5 to be positive for MWD ≤ 250 m, and patients with a simplified score of 2, 1, or 0 to be negative.

**Table 6:** Simple 1-digit score with binary questions.

During the last week, how difficult was it for you to walk 1 block (100 m), on level ground, without stopping to rest?	No difficulty, some difficulty, or slight difficulty	0
	Much difficulty/unable to do	2
What maximum distance can you walk before stopping because the pain becomes unbearable?	Answers strictly above 750 m	0
	Answers below 750 m	1
Compared to the usual walking speed of your relatives, friends, or people of your own age, do you think that you personally usually walk…	“A bit slower,” “same speed,” or “faster”	0
	“Much slower” or “moderately slower”	1
How long can you walk slowly (slower than the usual speed of relatives, friends, or other people of your own age) on level ground without stopping?	“1 hour” or “3 hours or more”	0
	“Not possible,” “30 seconds,” “1 minute,” “3 minutes,” “10 minutes,” or “30 minutes”	1
**Threshold for indication of walking** **≤250 m**	>2	
Area under the curve of simple 1-digit score	0.79 (0.74, 0.84)	
Accuracy	71.4% (66.3, 76.6%).	
Correlation coefficient	−0.52 (−0.60, −0.43).	

The area under the curve was 0.79 (0.74, 0.84) for the 1-digit score model. For the population, the sensitivity of the 1-digit model was 73.0% (66.6, 79.4%), specificity was 70.0% (62.7, 76.6%), and accuracy was 71.4% (66.3, 76.6%). The correlation coefficient representing the correlation between the simplified 1-digit score and MWD as measured by the treadmill test was −0.52 (−0.60, −0.43).

**Figure 2:**
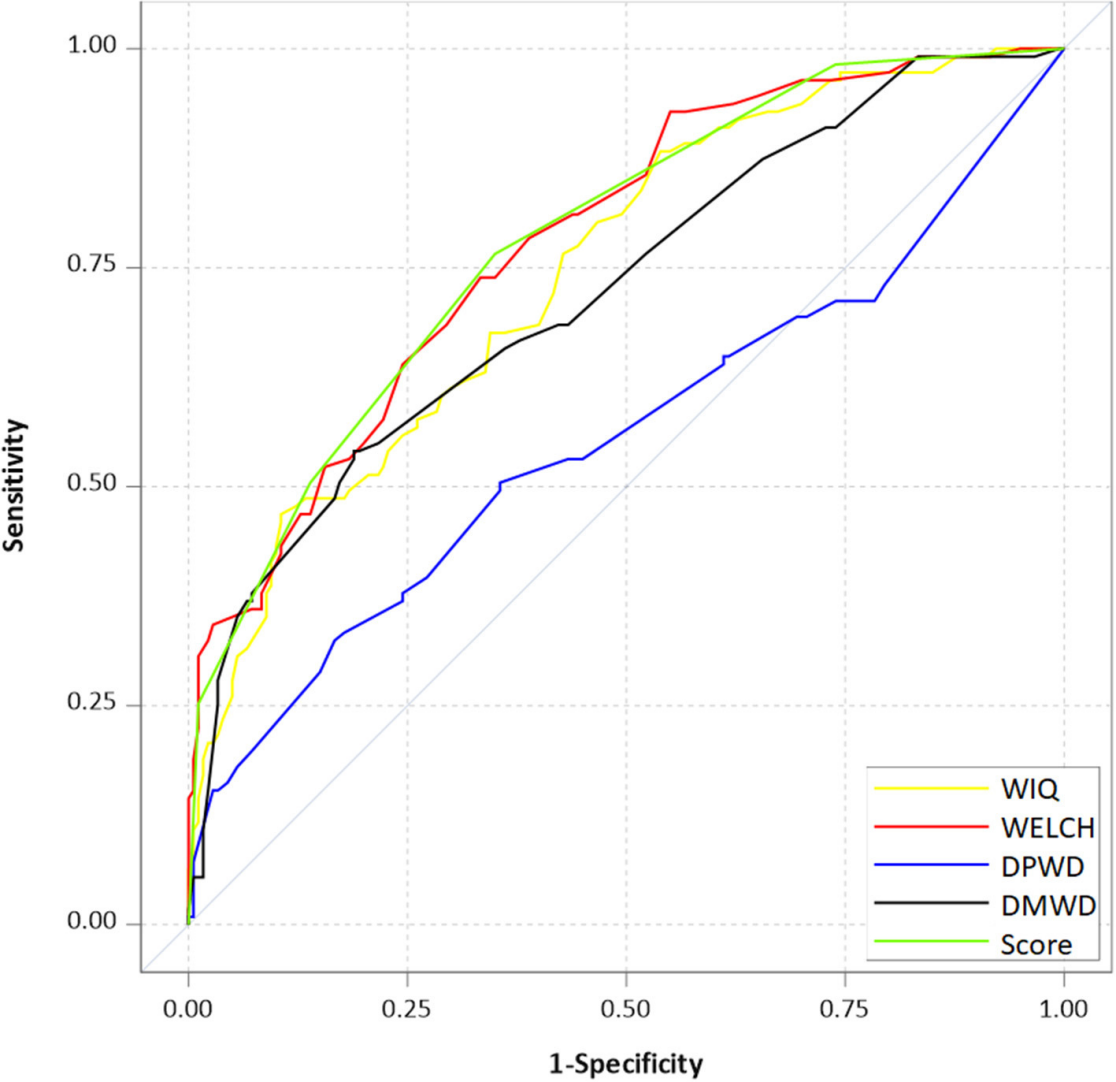
Receiver operating characteristic curves for questionnaires used to predict whether patients with LEAD can walk ≤ 250 m in a treadmill test. WIQ, Walking Impairment Questionnaire; WELCH, Walking Estimated-Limitation Calculated by History; DMWD, declared maximal walking distance; DPWD, declared pain-onset walking distance.

Receiver operating characteristic (ROC) curves are presented in Figure 2; these illustrate the fact that the simplified score can be as accurate as the WIQ and the WELCH.

## 4. Discussion

### 4.1. Main results

This study aimed to determine the threshold for scores on the WIQ and the WELCH that would best predict walking ≤ 250 m in a treadmill test (10%; 2 mph); to demonstrate that WIQ and WELCH scores are more accurate than patients' own estimates; and to construct a new scoring instrument based on the most discriminative elements among clinical variables and the items of each of these questionnaires.

The findings confirmed the threshold for WELCH score to predict whether a patient can walk 250 m or less. In a previous study, Abraham et al. found a cut-off of WELCH score equal to or lower than 25 to detect a walking distance of 257 m or less (8). In our study, with a threshold of 22, the measured sensitivity of the WELCH questionnaire was lower at 69.7% (63.1, 76.4%), but specificity was higher at 67.0% (59.8, 74.1%), consistent with a lower cut-off. We also identified a WIQ score threshold of 64% for prediction of a maximal walking distance of 250 m. This result is consistent with a previous study that found that an overall WIQ score of 42.5% or less identified low performers (i.e., LEAD patients with MWD < 160 m) with a sensitivity of 90% and specificity of 73% (13).

The results of the present study confirm the moderate correlation of WIQ score [0.51 (0.42, 0.59)] and WELCH score [0.55 (0.46, 0.62)] with MWD as assessed in the treadmill test in our population. Other clinical studies have found a “weak” correlation of these scores with objective measurement of walking distance, with correlation coefficients ranging from 0.33 (14) to 0.59 (15). The WIQ and the WELCH instruments were not constructed based on multivariate analysis to determine the questions of interest, and they were not designed to predict whether a patient can walk ≤ 250 m. Despite this, we found that the answers to WELCH and WIQ items were statistically associated with walking ≤ 250 m. In the present study, when clinical data were included in the model, only items taken from the WELCH questionnaire and the WIQ, along with the patient's declared maximal walking distance, remained in the model. This result is consistent with other studies, as the use of questions soliciting self-estimates of speed or distance is already known to improve the performance of the WIQ and the WELCH questionnaire (16).

Known factors for walking impairment did not appear as explanatory variables. This means that, although present tobacco consumption (17), absence of statin treatment (18), diabetes (19), female sex (20), and high body mass index (21) negatively influence walking distance, these factors fail to discriminate between patients who can walk ≤ 250 m and those who can walk further.

Our results show that the use of a simple 1-digit scoring model consisting of only four simple questions could enable accurate classification of patients in 73% of cases. These questions include the patient's own estimates, their average walking speed, the level of difficulty of walking 100 m at average speed, and the maximal duration of slow walking; they have only binary response options, as the options used in the WELCH questionnaire and the WIQ were regrouped. The 1-digit scoring model was significantly more accurate and more strongly correlated with the MWD than the patients' estimates. The correlation and accuracy of the 1-digit scoring model were similar to those of the WIQ and WELCH, but with a much simpler questionnaire. The use of existing questionnaires may also be helpful for further multicentric and international validation, as the WELCH and the WIQ each have validated versions in multiple languages.

### 4.2. Limitations

This study was retrospective. No external prospective validation study has been conducted with the simplified 4-item score. The questions were all presented as part of a questionnaire, with a logical path between questions, and results from the presentation of isolated questions might differ from results obtained from their presentation within the questionnaire. Therefore, we advocate against the use of the simplified 4-item score before external and internal validation. Moreover, the study was conducted at a single center, in the French language; it was also conducted by doctors familiar with the WIQ and the WELCH. We intend to conduct further research to investigate these issues and mitigate these limitations.

All variables were evaluated only against a treadmill test at 3.2 km/h and 10% grade. We do not know what the results would be if they were evaluated against other treadmill protocols or a 6-min assessment or real-life assessment of walking distance. Moreover, we did not investigate whether WIQ scores, WELCH scores, or scores on the simple 1-digit model would reflect any increase or decrease in walking distance over time or after an intervention with good sensitivity. For instance, in previous studies, changes after revascularization have tended to be overestimated by WIQ and WELCH scores, which decrease to a greater extent than the objective change observed in treadmill tests (22).

The absence from the model of known factors for walking impairment, such as diabetes, age, and treatment for current conditions, as explanatory variables could be partly due to our study design. Our study population mostly consisted of patients receiving intensive treatment in a referral center. The wider applicability of the results, especially to sub-groups such as elderly people, people with impaired cognition, obese people, or non-Caucasian groups, is questionable. The education levels of patients and data on their social status were not collected. The impact on the model of including age, ethnicity, and education could require additional research.

All questionnaires were administered by trained physicians. We did not investigate the role of care management nurses or whether the use of these scores can improve disease management. Communication between caregivers is known to be a key factor for disease management success (23).

Moreover, the definitions of dyslipidemia and hypertension that we used prevented us from drawing any conclusion regarding the effects of antihypertensive or lipid-lowering drugs.

A sensitivity analysis was conducted in which the response options for the WIQ and the WELCH questionnaire were regrouped in an alternative manner, with the option “unable to do” forming one group. The number of patients in the “unable to do” class was very small, ranging from 0 to 21, and the resulting scoring instrument was much more complicated, with seven different questions included. Interestingly, ABI was identified as an explanatory variable under that analysis.

We could not plot Doppler waveforms as relevant clinical data for the measurement of walking impairment, as these data were not available for all patients, although a study has shown that Doppler waveforms are associated with MWD (24). It remains to be examined whether the addition of this clinical measurement will improve the performance of this scoring model or not.

## 5. Conclusion

In conclusion, a WIQ score ≤ 64% or a WELCH score ≤ 22 could help to predict a maximal walking distance equal to or lower than 250 m in a standardized treadmill test. A simplified score could be used for rapid evaluation of a patient's walking distance in a population of patients with LEAD. However, dedicated studies are still required before this 4-item score can be put into practice or used to pre-select patients.

## Data availability statement

The raw data supporting the conclusions of this article will be made available by the authors, without undue reservation.

## Ethics statement

The studies involving human participants were reviewed and approved by Comité d'Ethique du Centre Hospitalier Universitaire de Rennes. The patients/participants provided their written informed consent to participate in this study.

## Author contributions

QT and GM conceived and designed the analysis. QT, GM, and AM collected the data. ALF contributed analysis tools and EL performed the analysis. QT and GM wrote the paper, which was reviewed by AM, EL, and ALF. All authors contributed to the article and approved the submitted version.

## Figures and Tables

**Table A1:** Distribution of scores on the 1-digit scoring instrument in the study population.

**Score**	**Treadmill walking distance > 250 m**	**Treadmill walking distance ≤ 250 m**	**Total**
5	3 (6%)	51 (94%)	54
4	1 (25%)	3 (75%)	4
3	30 (27%)	81 (73%)	111
2	43 (52%)	40 (48%)	83
1	26 (72%)	10 (28%)	36
0	9 (100%)	0 (0%)	9
Total	112	185	297
